# Systematic Identification of Novel Ferroptosis-Associated Multigene Models for Predicting Patient Prognosis Based on Endometrial Cancer

**DOI:** 10.1155/2023/4512698

**Published:** 2023-02-01

**Authors:** Xiqin Liu, Yingqi Lin, Xiaoling Liu, Xia Han, Jia Wu

**Affiliations:** ^1^Shenzhen Hospital of Guangzhou University of Chinese Medicine (Futian), Shenzhen, China; ^2^Guangzhou University of Chinese Medicine, Guangzhou, China; ^3^Zhongshan Hospital of Traditional Chinese Medicine, Zhongshan, Guangdong Province, China; ^4^Reproductive & Genetic Hospital of Citic-Xiangya, Changsha, Hunan Province, China

## Abstract

**Objective:**

Uterine corpus endometrial carcinoma (UCEC) is a frequent epithelial cancer in females. The rate of UCEC occurrence increases year by year and the age is getting younger and younger, which requires more active treatments to improve its prognosis. Ferroptosis is a kind of regulatory cell death that relies on iron and may be triggered by sorafenib, which has been elucidated in several cancers, but the mechanism of ferroptosis-related genes in UCEC has yet to be fully defined and will need more investigation.

**Methods:**

The mRNA expression profiles and accompanying clinical data of UCEC patients included in this research were obtained from a publicly available database. We subsequently classified the patients into experimental and training sets. Next, utilizing the least absolute shrinkage and selection operator (LASSO) Cox regression model, we established the multigene features of the TCGA experimental set and verified them in the validation set.

**Results:**

Per the findings of our investigation, the TCGA experimental set cohort had four differentially expressed genes (DEGs) that were linked to overall survival (OS). An analysis was conducted using univariate Cox regression (with all variables corrected for *P* < 0.05). To stratify the patients into two distinct categories, high- and low-risk, a diagnostic model premised on the identified four genes was formulated. In contrast with the low-risk population, the high-risk category exhibited a considerably lower OS (*P* < 0.0001). The findings of the multivariate Cox regression analysis illustrated that the risk score independently served as a predictor of OS (HR > 1, *P* < 0.01). The predictive capability of the model was verified by ROC curve analysis. Immune-related pathway enrichment was found using functional analysis, which illustrated that the two risk groups had significantly different immunological statuses.

**Conclusions:**

A unique model of genes linked to ferroptosis has the potential to be a treatment option for UCEC and can be utilized for the prognostic prediction of the disease.

## 1. Introduction

Uterine corpus endometrial carcinoma (UCEC) is an epithelial neoplasm that often develops in the endometrium of females. Globally, the incidence of this condition is rising in the reproductive system, while the average age at which it first appears is getting younger [1]. The stage of the illness as well as the specific histology of the tumor play a significant role in determining the prognosis for patients diagnosed with UCEC. In comparison, the five-year survival rate for stages I and II UCEC is around 80–90% and 70–80%, respectively, compared to the percentage of stage III and stage IV illness, which is 20–60% [2, 3]. Estimating the prognosis of UCEC has sparked the interest of numerous research studies in the past that have made use of biomarkers.

Ferroptosis is a type of regulatory cell death (RCD) that is reliant on iron and is caused by a potentially deadly buildup of lipid peroxidation [4, 5]. Recently, triggering ferroptosis in cancer cells has emerged as a potentially useful treatment strategy that induces death, particularly in the case of malignant tumors [6, 7] that do not respond to standard treatment. Tumor metabolism promotes immune evasion via inducing *T* cell dysfunction and exclusion [8, 9]. In addition to apoptosis and senescence, tumor cell ferroptosis is a previously unappreciated mechanism for *T*cell-mediated tumor clearance in vivo. Wang et al. [10] reported that cystine restriction may be a potential endogenous trigger for tumor cell ferroptosis in the tumor microenvironment, but the endogenous mechanism(s) triggering tumor cell ferroptosis remain to be defined in cancer patients. In addition to the mechanisms that are known to activate ferroptosis, a large number of genes that function as modulators or indicators of ferroptosis have been uncovered. P53 is the most studied oncogene and can promote or inhibit ferroptosis by regulating the transcription of its different target genes [11]. Researchers found that downregulation of SQLE are essential for p53-mediated tumor suppression [12]. The activation of SAT1 expression induces lipid peroxidation and sensitizes cells to undergo ferroptosis upon reactive oxygen species (ROS)-induced stress, which also leads to suppression of tumor growth in xenograft tumor models [13]. Furthermore, other genes such as GPX4 (GPX4) [14], nuclear factor erythroid 2-related factor 2 (Nrf2) [11], and NF-kB [15] are also reported to be closely associated with ferroptosis. Furthermore, researchers have reported that the process of ferroptosis was aberrantly regulated in UCEC cells, and ferroptosis activator can bring out cell death in UCEC cells [16, 17]. It is uncertain at this time, however, whether or not these ferroptosis-related genes (FRGs) have any link to UCEC patients' prognoses.

In this research, we started by collecting the mRNA expression patterns and the associated clinical data for UCEC patients from an online database that is accessible to the public. After that, we built a predictive polygenic signature of differentially expressed genes (DEGs) linked to ferroptosis in the TCGA cohort and verified the model. Lastly, we carried out a new prognostic model for four genes linked to ferroptosis. This model was demonstrated to be independently linked to OS in both the derived and validation set, which has implications for predicting UCEC prognoses.

## 2. Materials and Methods

### 2.1. Data Acquisition

RNA-Seq expression data and clinical follow-up data from UCEC patients were acquired from TCGA using TCGA bio-links, until March 10, 2021. We eliminated the samples that had no survival time or a survival time of 0. The scaling approach that was included in the “limma” R package was employed to conduct the normalization of the gene expression profiles. The ID was converted to gene symbol and we classified high grade to *G*3 category in clinical grade. The TCGA sample size included in the statistics was 539 and was separated into training and validation sets for subsequent subtype analysis and modeling analysis (Table 1). Next, sixty genes associated with ferroptosis were obtained from previously published research [5, 6, 18, 19]. This research did not need clearance from the local ethics committee since the TCGA data had already been made available to the public, and we followed the TCGA's data acquisition policy and publication guidelines.

### 2.2. Ferroptosis-Related Gene Features of Construction and Validation

With a false discovery rate (FDR) of <0.05, the “limma” R program was utilized to find DEGs in the TCGA cohort between tumor tissues compared to surrounding nontumor tissues. To search for ferroptosis-associated genes (FRGs) that have potential prognostic use, a univariate Cox analysis of OS was undertaken. Benjamini and Hochberg's (BH) approach was utilized to perform the corrections on the *P* values. Through the use of the STRING database (version 11.0) [20], interactive networks that overlap prognostic DEGs were produced. In developing the prognostic model, a Cox regression analysis of LASSO penalties was utilized to lower the likelihood of the model overfitting [21, 22]. We employed the LASSO technique, and the “glmnet” R package was utilized to conduct variable selection and shrinkage. The standardized expression matrix of the putative prognostic DEGs served as the regression's independent variables, and the OS and status of the patients included in the TCGA cohort served as the response variables in this study. By using ten-foldcross-validation and basing our decisions on the minimal criteria, we were able to establish the model's penalty parameters (*λ*) (i.e., the *λ* value corresponds to the minimum partial probability deviation). Patients' risk scores were determined by using the normalized expression levels of each gene in conjunction with the regression coefficients that corresponded to those levels. The equation may be established in the following way: score = *e* sum × corresponding coefficient for each gene.

Patients were classified into categories considered to be at high- and low-risk groupings predicated on the risk score median value. The “surv-cutpoint” function of the resultant version of the “survminer” R package was utilized for the purpose of calculating the optimum cuff-off expression value and performing a survival analysis on each gene that was expressed in the results. A time-dependent ROC curve analysis was performed utilizing the “survival ROC” in the R package to examine the predictive efficacy of gene features.

### 2.3. Functional Richness Analysis

The GO and KEGG analyses were executed according to the DEGs between the high- and low-risk groupings (|log 2FC| ≥ 1, FDR <0.05) using the “Cluster Profiler” included in *R*. The BH approach was used to make the relevant adjustments to the *P* value. Single-sample gene set enrichment analysis (ssGSEA) in the “gsva” *R* package [23] was employed to evaluate the stromal, immune, and ESTIMATE scores. The scores of 10 immune cells were examined utilizing the ESTIMATE function in *R* and the MCP counter, whereas the scores of 28 different immune cells were evaluated utilizing the GSVA package's ssGSEA technique [24].

### 2.4. Statistical Analysis

When comparing gene expression in tumor and neighboring nontumor tissues, the independent sample *t*-test was the statistical method of choice. The chi-square test was utilized to analyse the data and determine the significance of the proportional differences. To make a fair comparison between the high- and low-risk categories in terms of the ssGSEA scores of immune cells or pathways, the BH was utilized to adjust the *P* values via the Mann–Whitney test. The Kaplan–Meier analysis along with the log-rank test was implemented to contrast the OS across various groupings. To find independent determinants of OS, we conducted both univariate and multivariate Cox regression analyses. SPSS version 25.0 or R software, version 3.5.3, was utilized to execute all analyses of statistical data. In the absence of a specific statement to the contrary, the significance level was established at a *P* value <0.05 and all *P* values were two-tailed.

## 3. Results

### 3.1. Molecular Subtypes Were Identified Using a Consensus Clustering Algorithm

**Figure 1:**
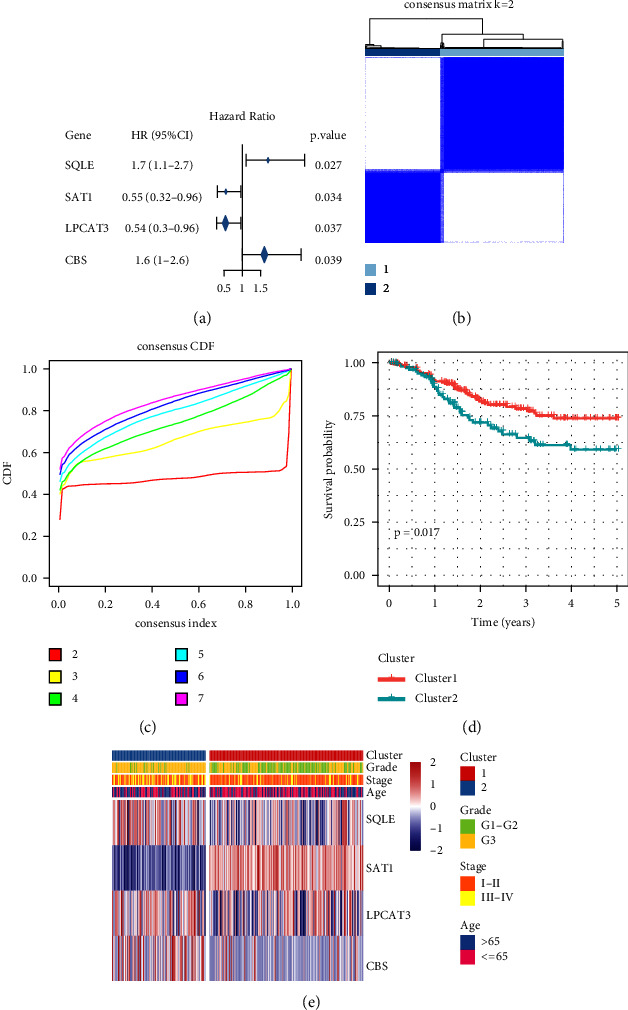
Determination of molecular subtypes utilizing consistent clustering algorithms. (a) Prognosis-related genetic forest maps. (b) Heatmap for consistent clustering *k* = 2. (c) Cumulative distribution of clustering consistency. (d) Time prognostic survival curves of different molecular subtypes of PFI. (e) Cluster heatmap of 4 prognostic-related genes.

The expression of 60 ferroptosis-related genes (FRGs) was derived from the TCGA training set expression profile data, and then one-way Cox analysis was undertaken with the aid of the coxph function included in the *R* program to acquire the four genes linked to UCEC prognosis (*P* < 0.05). The findings of the one-way analysis were used to guide the generation of a forest plot (Figure 1(a)). Consensus Cluster Plus was utilized to reliably cluster the four genes that proved to be significant in the univariate Cox analysis. These genes were clustered depending on their significance in the one-way Cox analysis. The km and Euclidean distances were employed as clustering algorithms and distance measures, correspondingly; with the threshold value of *k* = 2, the samples were able to be clustered together (Figure 1(b)). Prognostic ferroptosis-related gene expression in 2 subclasses (Figure 1(c)) shows remarkable differences (variations) in the expression levels of genes in *C*1 and *C*2. Subsequent investigation into the prognostic relationship (Figure 1(d)) between the two clusters illustrated statistically significant variations in *C*1 and *C*2. Additionally, substantial variations were seen in the clustering heatmap analysis of the four genes when comparing *C*1 and *C*2 (Figure 1(e)).

### 3.2. Determination of Differentially Expressed Genes

**Figure 2:**
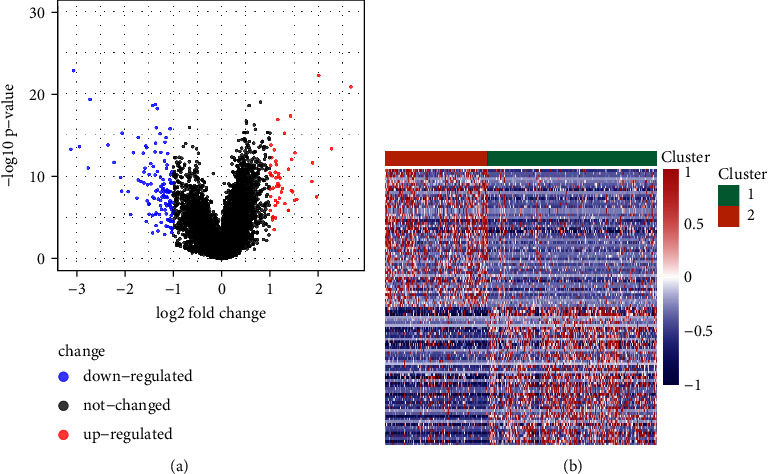
Differentially expressed gene identification. (a) Volcano map of differentially expressed genes between *C*1 and *C*2. (b) Heatmap of differentially expressed genes between *C*1 and *C*2.

DEGs between *C*1 and *C*2 molecular isotypes were identified separately with the limma package and screened predicated on the criteria of FDR <0.05 and |log 2FC| > 1. The detailed results are shown in a volcano map of DEGs with 52 upregulated genes (Figure 2(a)) and 125 downregulated genes and 50 upregulated and downregulated genes each, and a heatmap is shown in Figure 2(b).

### 3.3. Functional Analysis of Differentially Expressed Genes

**Figure 3:**
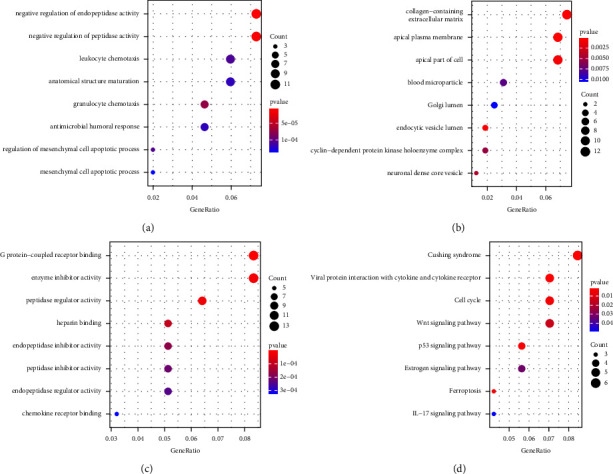
Functional analysis of differentially expressed genes. (a) BP annotation map for molecular subtypes of differentially expressed genes. (b) CC annotation molecular subtype of differentially expressed genes. (c) MF annotation diagram of molecular subtypes of differentially expressed genes. (d) KEGG annotation diagram of molecular subtypes of differentially expressed genes.

GO functional enrichment and KEGG pathway analyses of DEGs between C1 and C2 molecular isotypes were performed by the R package Cluster profiler. The GO functional annotation of DEGs with 373 entries enriched in BP (*P* < 0.05), top 8 for Figure 3(a); 22 to CC (*P* < 0.05), top 8 in Figure 3(b); and 49 enriched in MF (*P* < 0.05) in Figure 3(c). The KEGG pathway enrichment for differential genes (*P* < 0.05) resulted in 23 entries, which are shown in Figure 3(d). As shown in Figure 3(d), the eight of these pathways are viral protein crosstalk with cytokines and cytokine receptors, cushing syndrome, p53 signaling pathway, cell cycle, ferroptosis, Wnt signaling pathway, estrogen signaling pathway, and IL-17 signaling pathway.

### 3.4. Immune Score Comparisons between Different Molecular Subgroups

**Figure 4:**
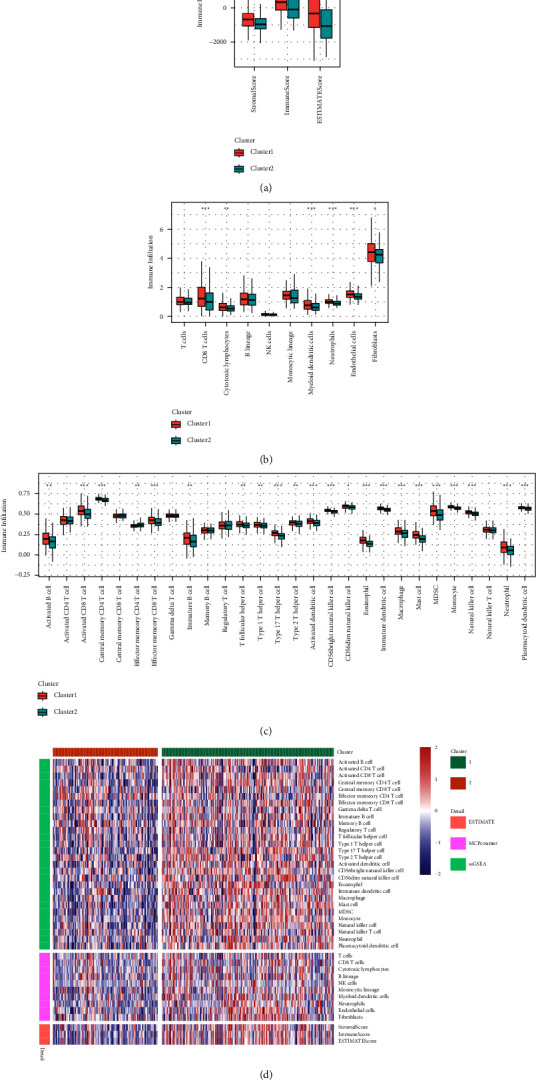
Comparative analysis of immune scores between molecular subgroups. (a) Analyses of differences in the estimated immune scores between molecular subgroups. (b) Evaluation of MCPcounter immune scores between molecular subforms. (c) Analyses of the variations in ssGSEA immune scores between molecular subforms. (d) A comparative assessment of the immunological scores across different molecular types using three different immune software programs.

To evaluate the association between immune scores of various molecular subgroups, the immune, stromal, and ESTIMATE scores as well as the scores of 10 immune cells were computed utilizing the ssGSEA approach of the GSVA package which was applied to 28 immune cells and the variations in immune scores between subgroups were compared (Figure 4). The ssGSEA results (Figure 4(a)) indicate that as opposed to *C*1, *C*2 had a significant elevation in the levels of *T* follicular helper cells, central memory CD4 *T* cell, activated CD8 *T* cells, effector memory CD8 *T* cells, activated *B* cells, Type 17 *T* helper cells, Type 2 *T* helper cells, Type 1 *T* helper cells, immature *B* cells, CD56dim natural killer cells, MDSC, immature dendritic cells, macrophages, CD56bright natural killer cells, mast cells, activated dendritic cell, neutrophils, natural killer cells, monocytes, eosinophils, and immune score for the plasmacytoid dendritic cells. In addition, the *C*1 had a significantly higher immune score for the effector memory CD4 *T* cells in contrast with the *C*2. The MCPcounter results (Figure 4(b)) indicate that, as opposed to *C*2, *C*1 exhibited a significant elevation in the levels of neutrophils, myeloid dendritic cells, endothelial cells, cytotoxic lymphocytes, CD8 *T* cells, and immune score for the fibroblasts. The estimate results (Figure 4(c)) indicate that in contrast with *C*2, *C*1 has significantly higher stromal, immune, and ESTIMATE scores. In addition to this, we evaluated the immune scores of three different immune software programs across different molecular subsets (Figure 4(d)), which revealed significant variations.

### 3.5. Development of a Prognostic Risk Model Premised on Module Genes

**Figure 5:**
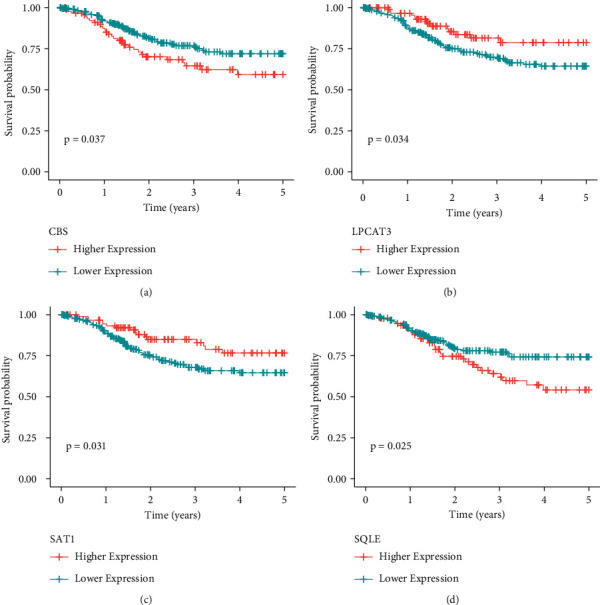
The KM curves of the 4 genes (on the TCGA training set).

**Figure 6:**
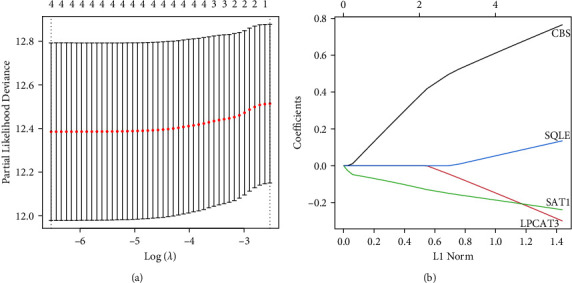
LASSO analysis of the 4 genes. (a) The trajectories of all of the independent variables, with the logarithmic value of each independent variable lambda illustrated along the horizontal axis. The graph's vertical axis shows the coefficient of the independent variable. (b) The confidence interval for each lambda.

Using the data from the training set data, an FRG expression matrix was established based on the expression matrix as well as survival data, the univariate Cox analysis utilizing the coxph function of the *R* package survival, and *P* < 0.05 as the threshold to filter the 4 genes related to prognosis, as illustrated in Figures 5(a)–5(d). Further LASSO analysis was conducted for these four genes. The LASSO (least absolute shrinkage and selection operator) technique is a compressed estimate that yields a highly accurate model via the formulation of a penalty function, in such a way that it may condense specific coefficients whilst at the same time adjusting some other values to zero. Thus, while retaining the advantage of subset shrinking, a biased estimate approach with complicated collinearity data may realize the choice of variables when conducting parameter estimation and effectively tackle the multicollinearity issue in regression analysis. As part of our investigation, we worked with the glmnet function in the *R* program to carry out LASSO Cox regression. As can be seen in Figure 6(a), the initial step included analyzing the change trajectories for every independent variable. The number of independent variables that had coefficients that were approaching zero likewise progressively rose as the lambda steadily increased. Model construction was conducted by means of ten-foldcross-validation.  Figure 6(b) presents the results of an investigation of the confidence intervals for each lambda. When lambda equals 0.001460945, the value is at its most desirable level. As a result, four genes at lambda = 0.001460945 (CBS, LPCAT3, SAT1, and SQLE) were chosen as the target genes for the next step.  Figure 5 portrays the prognostic KM curves for the four distinct genes. The resultant formula for the 4-gene signature is shown below. Risk score = CBS *∗* 0.77 + LPCAT3 *∗* −0.3 + SAT1 *∗* −0.24 + SQLE *∗* 0.14.

### 3.6. Development and Assessment of the Risk Models

**Figure 7:**
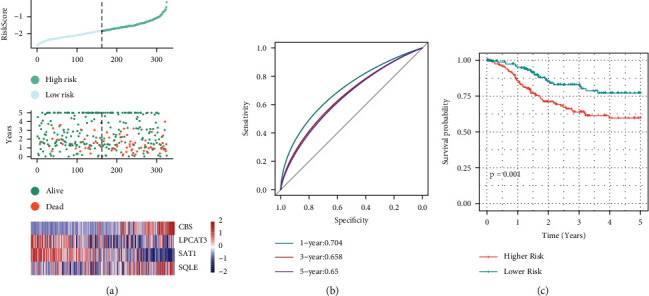
Calculation of the risk scores for each sample (on the TCGA training set). (a) Risk score, survival time, survival state, and 4-genes expression in the training set. (b) ROC curve for classifying 4-gene signatures. (c) The distribution of the KM survival curve of a 4-gene signature in the training set.

The expression level of the sample was taken into consideration while determining the risk scores for each individual sample. As illustrated in Figure 7(a), the distribution of risk scores for the samples has also been plotted. The heatmap illustrates the variation in the expression of four distinct signature genes associated with elevated risk levels. The findings highlight that the rate of deaths that occurred in the samples with higher risk scores was noticeably elevated in contrast to that of those with lower scores, suggesting that higher risk scores were linked to unfavorable prognoses. With the aid of the *R* package, further ROC analysis of prognosis stratification was carried out, and the effectiveness of prognostic prediction stratification over one, three, and five years were studied. As can be seen in Figure 7(b), the model has a large AUC area. Lastly, we distributed the samples into groups as per the risk score. Samples with a score above the median were allotted to the high-risk group, while those having a score below the median were assigned to the low-risk group. A remarkable variation could be seen in the KM curve, as illustrated in Figure 7(c) (*P*=0.001). Then, the 162 samples were grouped under the high-risk groups, and the other 163 samples were allocated to the low-risk groups.

### 3.7. Verification of the Reliability of the 4-Gene Signature Using an in-House Dataset for the Risk Model

**Figure 8:**
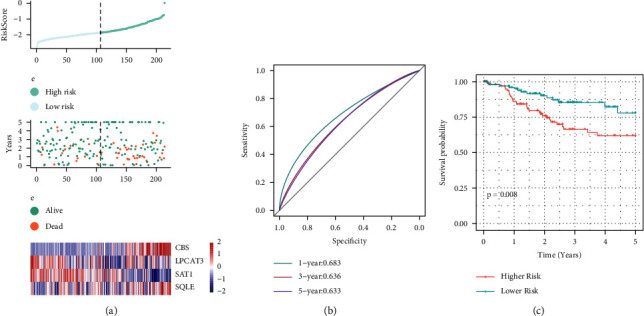
Calculation of the risk scores for each sample (on the TCGA testing set). (a) The risk score, survival time, survival status, and 4-gene expression of the validation set. (b) ROC curves for 4-gene signature classification. (c) Distribution of 4-gene signature KM survival curves in the test set.

We employed a similar model and coefficients comparable to the ones in the training set and the whole data set to assess the model's robustness. We then computed each sample's risk score predicated on their expression levels and charted the distribution of the risk scores for these samples.  Figure 8(a) depicts the risk score distribution that was obtained from the validation set which illustrated statistically significant variation between the proportion of dead samples having higher risk scores and those having lower risk scores, implying that higher risk scores were linked to unfavorable prognosis. With the help of the pROC package in *R*, we performed a ROC analysis of the prognostic stratification. One-year, three-year, and five-year outcomes are shown in Figure 8(b). The AUC area of the model is rather high. Lastly, we classified the samples premised on the risk scores. The samples that were less than the median were considered to be in the low-risk category, while those that were over it were in the high-risk population. After plotting the KM curve as depicted in Figure 8(c), we observed significant variations (*P*=0.008). Of these, 107 samples were classified into the high-risk category, and 107 samples were determined to belong to the low-risk category.

**Figure 9:**
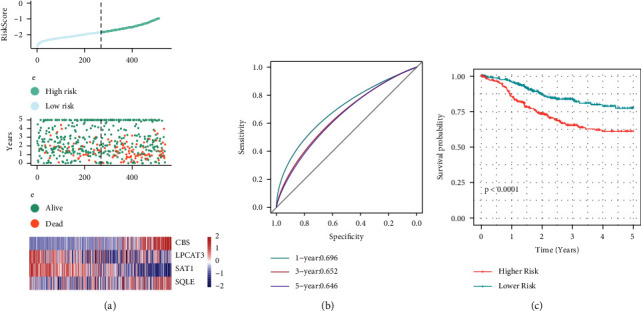
Calculation of each sample's risk scores (on the full TCGA set). (a) Risk score, survival status, survival time, and 4-gene expression in the whole dataset. (b) ROC curves for classifying 4-gene signatures across the entire dataset. (c) KM survival curves for 4-gene signature in the whole dataset.

Figure 9(a) depicts the distribution of risk scores over the whole dataset. The proportion of dead samples associated with higher risk scores was considerably elevated in contrast with that associated with lower scores, which demonstrates that higher risk score samples had a direr prognosis. Furthermore, with the *R* software package (pROC), we conducted a study of the ROC curve to ascertain the prognostic stratification of the risk score.  Figure 9(b) depicts the results of an examination of the one-, three-, and five-year prognoses, correspondingly. The AUC area of the model is rather high. Afterward, we grouped the samples based on the risk score, whereby those exceeding the median were assigned to the high-risk subgroup, while those less than the median were placed under the low-risk subgroups. As displayed in Figure 9(c), the plotted KM curve illustrated extremely significant differences (*P* < 0.0001), of these, 269 and 270 samples were classified into the high- and low-risk groupings, respectively.

### 3.8. Risk Model and Prognosis Analyzes of Clinical Parameters

**Figure 10:**
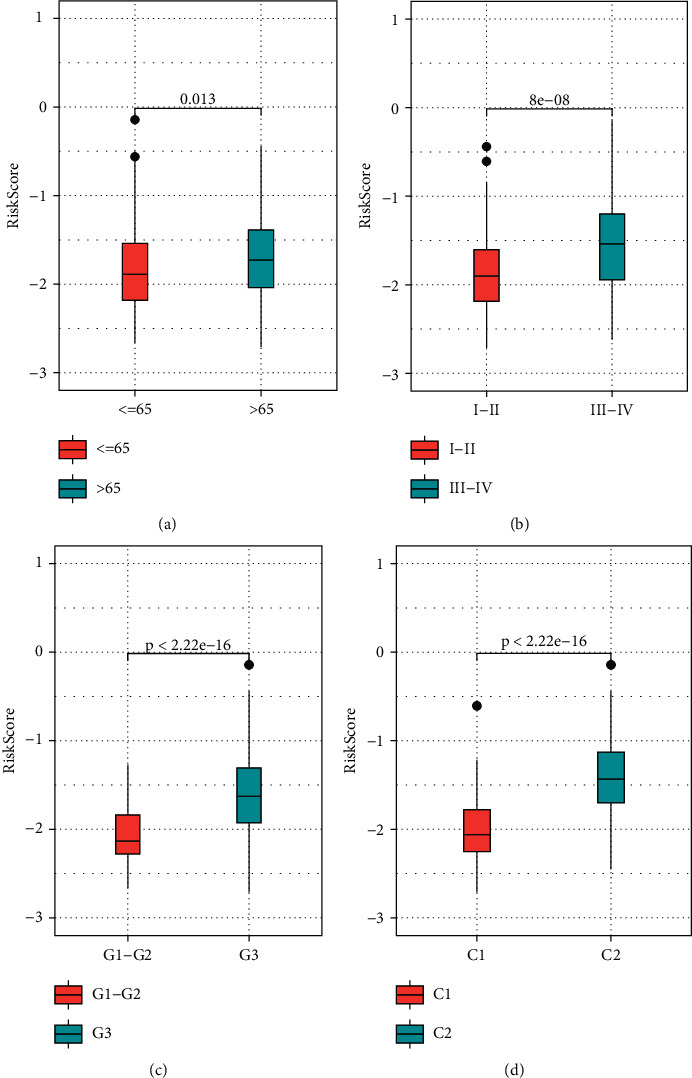
Differences in risk scores among molecular subtypes. (a) Risk score comparisons between age-grouped samples. (b) Risk score between stage-grouped samples. (c) Risk score between grade-grouped samples. (d) Risk score between molecular subtype samples.

**Figure 11:**
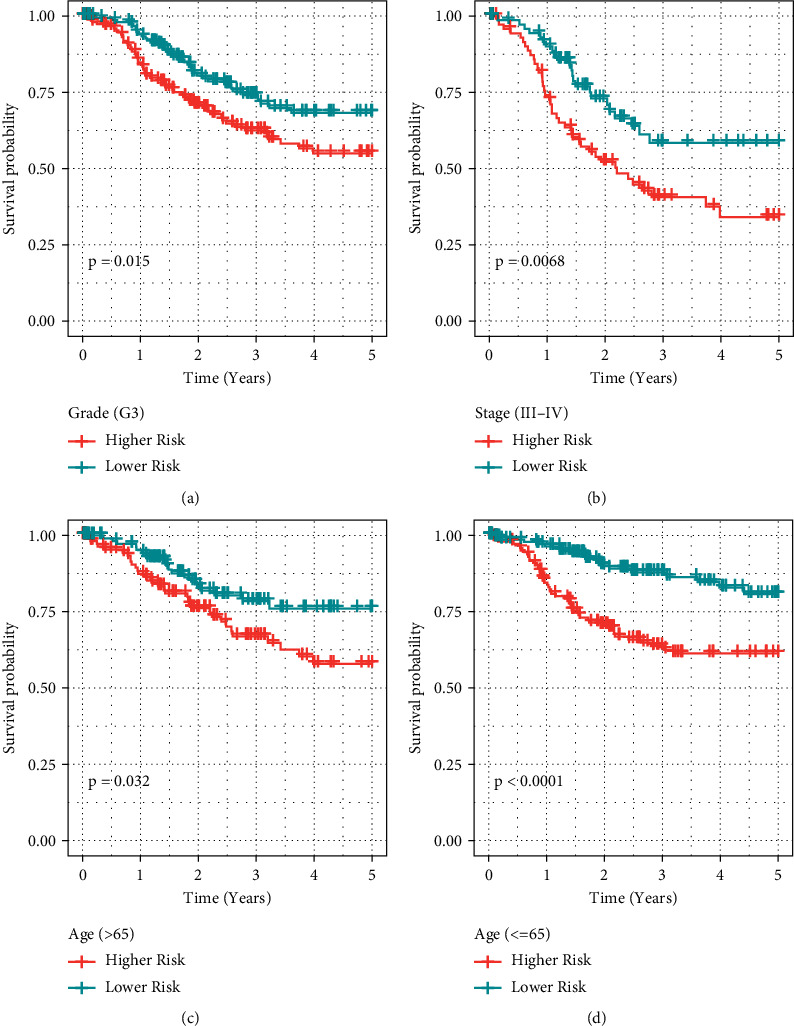
RiskScore could effectively differentiate between high- and low-risk categories for grade *G*3, stage III-IV, >65 years, and <65 years for multiple clinical characteristics. (a) Survival study of *G*3 patients in the high-risk category. (b) Survival study of high-risk patients in stages III-IV. (c) Survival analysis of patients aged >65 years in high- and low-risk populations. (d) Survival analysis of patients aged <65 years in high- and low-risk populations.

Subsequent risk score analysis of 4 genes found that risk scores scored higher in stage III-IV in patients older than 65 years and were significantly different in grade *G*3 patients. When we evaluated the variations in the risk scores of the various molecular subtypes, we found that the prognosis of patients with the *C*2 subtype had significantly higher risk scores in contrast with those with *C*1 subtypes (Figures 10(a)–10(d)). The finding indicated the risk score was able to differentiate between grade *G*3, stage III-IV, >65 years, and <65 years when various clinical variables substantially differed between high- and low-risk populations (Figures 11(a)–11(d)), which offered additional support that our model possesses potent predictive performance in various clinical parameters.

### 3.9. Univariate and Multivariate Analyses of the 4-Gene Signature

To ascertain if the 4-gene signature model might be used independently in clinical settings, data on age, grade, stage, and risk score (Table 2) were systematically analyzed. We subjected the whole set of clinical data to an integrated complete data Cox regression analysis and utilized the univariate and multivariate Cox regression analyses to examine the applicable HR, 95% CI of HR, and *P* value. In both the univariate and the multivariate analyses, we observed that there was a substantial link between survival and the risk score as well as the stage. These data illustrate that our model 4-gene signature has strong prediction ability when it is applied to clinical settings.

**Figure 12:**
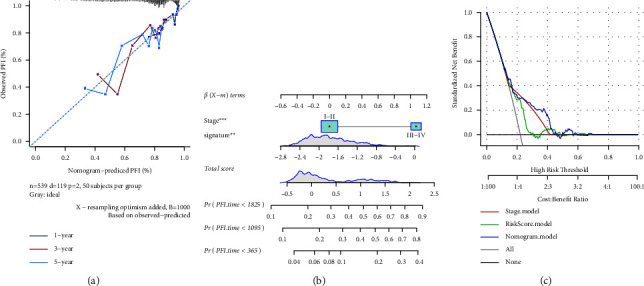
Univariate and multifactorial analyses of 4-gene signature. (a) Calibration curves over one, three, and five years for the column line graphs. (b) Column line diagram model for TNM_stage, tumor_stage, risk score, and age. (c) DCA curves for TNM_stage, tumor_stage, risk score, and age.

Mapping of the calibration curves, nomogram, and DCA (decision curve analysis) curves was conducted predicated on the two variables of risk score and stage, which are illustrated in Figures 12(a)–12(c).

### 3.10. Relationship between Risk Score and the KEGG Pathways

**Figure 13:**
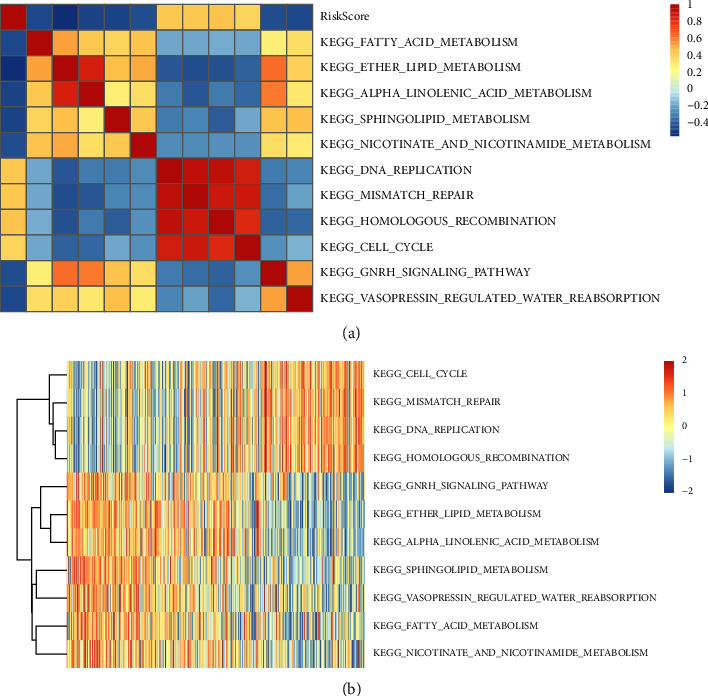
Association of risk score with the KEGG pathways. (a) Correlation coefficient clustering between KEGG pathways with correlations >0.4 and *P* < 0.05 with risk scores and between risk score. (b) Correlation with risk scores >0.4 and *P* < 0.05 for the KEGG pathway with increasing risk scores in each sample. The horizontal axis denotes the samples, whereas increasing risk scores move from left to right.

To ascertain the association of risk scores with biological function across a variety of samples, we employed the *R* package GSVA to choose the gene expression patterns that correspond to these samples for single-sample GSEA. After doing so, we computed each sample's score and assigned the ssGSEA score that corresponds to each function. Following additional analysis of the association of these functions with risk scores, it was determined that the absolute value of the Pearson correlation coefficient was >0.4, and the KEGG pathway that had a *P* value of <0.05 is depicted in Figure 13(a). There was a positive correlation between four of these pathways and the risk scores, whereas seven of these pathways had an inverse relationship with the risk scores. As depicted in Figure 13(b), the heatmap of 11 KEGG pathways was displayed depending on their enrichment scores. We found that the enrichment scores of CELL_CYCLE, HOMOLOGOUS_RECOMBINATION, MISMATCH_REPAIR, and DNA_REPLICATION increased with increasing RiskScore, whereas the enrichment score of SPHINGOLIPID_METABOLISM, ALPHA_LINOLENIC_ACID_METABOLISM, ETHER_LIPID_METABOLISM, FATTY_ACID_METABOLISM, NICOTINATE_AND_N‐ICOTINAMIDE_METABOLISM, GNRH_SIGNALING_P‐ATHWAY, and VASOPRESSIN_REGULATED_WAT‐ER_REABSORPTION decreased with increasing risk score.

## 4. Discussion

Ferroptosis is a new type of programmed cell death that functions as an interface between the ferritin gene and cancer therapy and prognoses. Ferphotography has garnered a lot of interest as a result of its possible antitumor capability. According to the findings of Xu et al. [25], the three primary links that contribute to iron poisoning are the dependence on Fe^2+^ (which supplies an electron for the oxidation reaction), the GPX oxidation process, GPX4 inactivation (an imbalance of antioxidants), and ROS production (primarily as a result of the Fenton reaction). The process of ferroptosis is a cascade that involves multiple enzymes, and it has been shown that several FR genes perform a crucial function in the development of malignancies. In this work, we carried out a comprehensive evaluation of the expression of sixty genes relevant to ferroptosis in UCEC tumor tissue, and we analyzed how these genes are associated with OS. We discovered that half of the FRGs was linked to OS, and the differential expression of FRGs was seen between the tumor and surrounding nontumor tissues in 70.6% of the cases. These data strongly imply the likelihood of a function for iron mortality in UCEC, as well as the feasibility of developing prognostic models using iron mortality-related genes. Therefore, we attempted to construct a new ferroptosis model by LASSO regression analysis with a prognostic model consisting of four ferroptosis disease-associated genes (SQLE, SAT1, LPCAT 3, and CBS). The four genes may be generally sorted into three distinct types, notably, lipid metabolism (SQLE and SAT1), iron metabolism (LPCAT 3), and energy metabolism (CBS) [6]. LPCAT3 is an enzyme that converts lytic phosphatidylcholine to phosphatidylcholine in the liver. It also maintains systemic balance and is involved in phospholipid remodeling, the growth of intestinal stem cells, and tumorigenesis [26, 27]. Cholesterol esterification mediated by SAT1 alleviates the inhibitory effect of SREBPs. SAT1 is a crucial intrinsic driver of cholesterol metabolism, and it performs an indispensable function in lowering the amount of cholesterol found inside cells, which helps to counteract the inhibiting impact of SREBPs [28]. Squalene epoxidase (SQLE) is an essential enzyme that regulates the rate of cholesterol production. The cBioPortal for Cancer Genomics Gene copy number or expression amount [29, 30] of cholesterol synthesis genes analyzed in The Cancer Genome Atlas (TCGA) cohort showed that SQLE is among the genes that are remarkably elevated in expression in the majority of cancers. There is mounting support for the hypothesis that a positive correlation exists between high levels of SQLE expression and a dismal prognosis in a variety of tumor types [31]. CBS is a pyridoxal 50-phosphate (PLP) enzyme that catalyzes the conversion of homocysteine (Hcy) and serine into cysteine by the transsulfuration pathway and regulates the metabolism of hydrogen sulfide (H_2_S), with higher H_2_S levels related to the proliferation of multiple tumor types [32]. In this study, the expression levels of these genes were shown to be elevated in UCEC tissues and had an unfavorable association with patients' prognoses. Because only limited relevant research data on these have been published on genes other than LPCAT3 and CBS, it is not yet known whether or not these genes are implicated in UCEC patients' prognoses by affecting the process of ferroptosis illness. Although the mechanism of tumor sensitivity to ferroptosis illness has been the subject of much inquiry over the last several years, the possible link between tumor immunity and ferroptosis disease is yet to be clarified. We conducted a GO analysis premised on the DEGs across the various risk groups and discovered that a great number of biological processes and pathways associated with the immune system were enriched. There is evidence to support the hypothesis that ferroptosis illness may have some connection to tumor immunity. Our research contains several noteworthy caveats. First, we generated and verified our prognostic model retrospectively by utilizing data from public sources. To prove its therapeutic value, additional analysis of prospective data will be required. Second, it is unavoidable to merely evaluate a single marker to show the inherent flaws of the prognosis model, since several important prognostic genes in UCEC could be excluded. Furthermore, it is important to note that there has been no conclusive experimental work done on UCEC to address the relationship between risk scores and immunological activation.

## 5. Conclusions

In this work, we describe a new prognostic model for four genes linked to ferroptosis. This model was demonstrated to be independently linked to OS in both the derived and validation sets, which has implications for predicting UCEC prognoses. Nonetheless, the exact mechanisms behind FRGs in UCEC and tumor immunity are not well known at this time and should be the subject of additional research.

## Figures and Tables

**Table 1:** The 4-gene signature model in clinical application, information on age, grade, stage as well as risk score.

Variables	Univariable analysis				Multivariable analysis			
	HR	HR. lower	HR. upper	*P* values	HR	HR. lower	HR. upper	*P* values
Age								
(>65 vs. ≤65)	1.1	0.75	1.6	6.80*E* − 01	0.99	0.69	1.4	9.70*E* − 01
Stage								
(III-IV vs. I-II)	3.5	2.4	5	**1.30*E* − 11**	2.8	1.9	4.2	**8.60*E* − 08**
Grade								
(*G*3 vs. *G*1-*G*2)	2.1	1.4	3.1	**5.40*E* − 04**	1.4	0.84	2.2	2.10*E* − 01
Risk score	2.6	1.8	3.7	**4.50*E* − 07**	1.6	1.1	2.4	**2.90*E* − 02**

The bold values represents a significant difference in the COX regression analysis. As a result, we can found that risk score and stage were significantly associated with survival in both univariate analysis and multifactor analysis.

**Table 2:** Group results of all the patients in this study.

Clinical features	TCGA total	TCGA test	TCGA train	*P* values
Age				
≤65	304	114	190	0.271
>65	235	100	135	
Stage				
I-II	387	151	236	0.674
III-IV	152	63	89	
Grade				
G1-G2	218	94	124	0.213
G3	321	120	201	
PFI				
0	415	168	247	0.568
1	124	46	78	

## Data Availability

This research study examined publicly accessible datasets. The following website has this information: https://www.cancer.gov/about-nci/organization/ccg/research/structural-genomics/tcga. Datasets were derived or evaluated in this study. All data are presented in the article and accompanying documents.
